# Supplementary material for: Time-dependent memory and individual variation in Arctic brown bears (Ursus arctos)
Source: Mov Ecol. 2022 Apr 11;10:18. doi: 10.1186/s40462-022-00319-4 (PMC8996616; doi:10.1186/s40462-022-00319-4)
Supplement: Supplementary file 1 — Additional file 1. Appendix providing additional modelling details along with tables that further characterize our results. [file 40462_2022_319_MOESM1_ESM.pdf]

Appendix: Time-dependent memory and individual variation in Arctic brown bears (*Ursus arctos*)

Peter R. Thompson[1,*], Mark A. Lewis[1,2], Mark A. Edwards[3,4], and Andrew E. Derocher[1]

[1]Department of Biological Sciences, University of Alberta, Edmonton, AB, Canada
[2]Department of Mathematical and Statistical Sciences, University of Alberta, Edmonton, AB, Canada
[3]Mammalogy Department, Royal Alberta Museum, Edmonton, AB, Canada
[4]Department of Renewable Resources, University of Alberta, Edmonton, AB, Canada
[*]Correspondence: pt1@ualberta.ca

1 Appendix: Supplementary Material

1.1 Additional modelling details

We applied a discrete-time movement model developed by Thompson et al. (2021) that allows for inference on animal behaviour and cognition. We fit the model to data using maximum likelihood estimation, calculating the likelihood function as the product of individual likelihoods for each point (Thompson et al., 2021). The probability of taking a step is equal to the probability of taking that step from the non-stationary state (f_{ns}) multiplied by the probability of being in the non-stationary state, plus the the probability of taking that step from the stationary state (f_s) multiplied by the probability of being in the stationary state. Multiplying these probabilities together for each step yields the probability that the animal would produce a track exactly like the observed data under a given set of model parameters (Whoriskey et al., 2017). We optimized the negative logarithm of this function to maximize numerical precision, since these probabilities are always very, very small numbers.

We fixed the conditional likelihood function for the stationary state, f_s, to remedy the model of additional complexity (Thompson et al., 2021). We assumed that the animal is not influenced by resources or memory in this state, as most of the "movement" we observe in the stationary state arises from GPS error anyway. We assume that when in the stationary state, the animal's turning angles follow a uniform distribution and its step lengths follow a half-Gaussian distribution:

$$f_s(\mathbf{x}_t, \phi_t | \mathbf{x}_{t-1}, \phi_{t-1}, \rho_s) = \left[\frac{1}{2\pi}\right] \frac{2}{\pi \rho_s} \exp - \frac{\|\mathbf{x}_t - \mathbf{x}_{t-1}\|^2}{\pi \rho_s^2}. \quad (1)$$

Here we fixed ρ_s such that the average step length was 30 m long, equal to the length of one grid cell from our environmental data.

We used automatic differentiation, via the Template Model Builder R package (Kristensen et al., 2016), to accurately fit the model amid high numerical complexity. For multidimensional functions such as ours, optimization routines typically require the calculation of the function's gradient. When the gradient of a function is 0 in every direction, there is potential that the optimizer has found a local optimum of that function. Traditional optimization does not require the user to provide a gradient along with their objective function, instead approximating the gradient at each point using finite-difference methods. Providing a gradient up front is faster computationally but difficult if there is no analytical expression for the gradient (as in most likelihood functions). TMB uses a pseudo-analytical automatic differentiation algorithm to calculate the gradient of a multidimensional likelihood function, alleviating the need for finite-difference approximation (Kristensen et al., 2016). While the negative log-likelihood function and its gradient are returned from TMB as R functions, the computational work is executed in C++, which also speeds up the optimization (Kristensen et al., 2016). We used the R *nlminb* function to optimize the appropriate TMB function for each model.

To prevent our model from producing errors or unrealistic results, we imposed bounds on some of the parameters. We bounded the estimation for μ at 365 days, the amount of GPS data we set aside for "training" the bears' memory. If μ were to be greater than this value, there would not be a testable memory signal for some of the data (i.e., we would not always know where the bear was μ days before). To identify potential signals greater than 365 days, we would have to sacrifice bears with insufficient data, thus decreasing our "population size" and our statistical power. We performed the optimization routine twice for each bear with different "initial values" for μ, since there was potential for local optima along the axis of this parameter (Thompson et al., 2021). We applied lower optimization bounds for μ and σ of 5 and 18 GPS fixes (20 hours and 3 days, respectively). When $\sigma < 3$ days, the partial derivative of our likelihood function with respect to μ became noisy, leading to computational errors in optimization. We additionally required $\alpha < -\log_{10} \frac{30}{1000} \approx 1.52$. We chose this bound because each grid cell is $\frac{30}{1000}$ km in length. For parameters with fairly restrictive bounds (λ, γ, β_d, and β_0, which are bounded between 0 and 1), we performed logit transformations ($\tilde{\lambda} = \log \frac{1}{1-\lambda}$, for example) so the optimizer would more effectively traverse the parameter space.

Instead of using traditional Wald-type confidence intervals for our parameters, we found them explicitly using likelihood profiling techniques (Fischer and Lewis, 2020). The algorithm used here finds confidence intervals for one parameter at a time by performing a binary search algorithm for a target function value (the optimal function value minus a confidence threshold of 1.96). The algorithm searches for each parameter by moving away from the optimal value in parameter space, fixing that parameter and optimizing the other ones. This process goes on until the algorithm gets close enough to the target function value. This means that, unlike traditional confidence intervals, the lower confidence bound may be farther away from the parameter estimate than the upper confidence bound.

1.2 Generating the berry density raster

We generated a vegetation class raster using the decision tree from Ducks Unlimited (2002) to classify each 30 x 30 m grid cell in the Mackenzie Delta region into one of 46 classes. Table S1 describes each class as well as its value for the berry probability raster used in the model.

1.3 Supplementary tables

Description	Berry probability	Percent of landscape
Open needleleaf	0.5	0.397
Woodland needleleaf (other)	1	1.481
Woodland needleleaf (moss)	1	0.303
Woodland needleleaf (lichen)	1	0.524
Closed deciduous	0.5	0.082
Open deciduous	0.5	0.344
Closed tall shrub	0	1.476
Open tall shrub	0	0.007
Medium willow shrub	0	0.480
Medium-tall willow shrub	0	0.356
Medium-tall shrub (other)	0	0.027
Low shrub (other)	0	14.087
Low shrub (recently burned)	0	0.352
Low shrub (wet)	0	0.004
Low shrub (floodplain)	0	0.688
Low shrub (delta lowlands)	0	1.243
Low shrub - wet graminoid	0	0.012
Low shrub - tussock	0	1.135
Low shrub (upland)	0.5	0.368
Dwarf shrub (other)	1	9.097
Dwarf shrub (tussock)	0	5.331
Dwarf shrub (lichen)	1	2.239
Dwarf shrub (unknown - Kendall area)	0.5	0.046
Dwarf shrub (tussock/Dryas)	0	0.072
Dwarf shrub (Dryas/heather)	0.5	0.796
Dwarf shrub (sloped hummocks)	0	0.035
Dwarf shrub (wet graminoid)	0.5	0.160
Tussock tundra	0.5	1.407
Lichen	0.5	0.243
Wet graminoid (wetland depressions or lake edges)	0	1.762
Wet graminoid (northern delta)	0	1.429
Wet graminoid (floodplain)	0	1.101
Wet graminoid (some shrubs)	0	0.111
Wet graminoid (unknown)	0	0.003
Dwarf shrub mosaic (wet)	0.5	0.355
Dwarf shrub mosaic (very wet)	0.5	0.761
Aquatic bed	0	0.014
Emergent vegetation	0	1.002
Emergent and other wet wetland areas	0	1.424
Clear water	0	20.108
Turbid water	0	25.385
Sparse vegetation	0	0.394
Sparse / non-vegetated (unsure)	0	0.030
Non-vegetated	0	0.670
Other	0	0.001
Unclassified	0	2.655

Table S1: Classification method used to generate the berry density raster for the resource-only and resource-memory models. The name of each original vegetation class is shown along with the assigned probability, representing how correlated each habitat type is to the presence of berries, as well as the percentage of the landscape that is covered by each class.

ID	Model	ρ_{ns}	κ	β_1	β_2	β_3	β_4	β_5	β_6	β_0	β_d	μ	σ	λ	γ	α
GF1004	RM	0.357	0.403	0.113	-0.031	0.707	-0.019	-0.055	-0.111	0.1068	∼1	350.6	9.1	0.460	0.729	-0.046
GM1046	RM	0.399	0.414	0.381	-1.341	0.732	-0.029	-0.015	-0.074	0.2011	0.7800	354.2	8.1	0.332	0.741	-0.259
GF1008	R	0.414	0.422	0.098	-0.885	1.536	-0.044	-0.127	-0.157	N/A	0	N/A	N/A	0.464	0.825	N/A
GF1086	RM	0.205	0.131	0.281	-1.307	2.579	-0.198	-0.006	-0.307	∼0	0.9792	0.8	3.0	0.346	0.739	-4.617
GF1016	M	0.279	0.110	0	0	0	0	0	0	0.7311	0.9354	1.4	3.0	0.549	0.722	0.072
GF1041	R	0.376	0.465	0.165	-1.450	2.497	-0.030	-0.275	-0.269	N/A	0	N/A	N/A	0.338	0.752	N/A
GF1107	R	0.236	0.141	0.222	-0.718	2.433	-0.020	-0.311	-0.241	N/A	0	N/A	N/A	0.387	0.712	N/A
GF1130	N	0.394	0.400	0	0	0	0	0	0	N/A	0	N/A	N/A	0.421	0.728	N/A
GF1005	R	0.597	0.202	0.158	-0.926	3.538	-0.060	-0.169	-0.153	N/A	0	N/A	N/A	0.404	0.716	N/A
GF1096	RM	0.366	0.273	0.207	-2.017	1.970	-0.040	-0.132	-0.188	0.9997	0.5002	337.0	16.4	0.330	0.755	-0.129
GF1167	M	0.420	0.427	0	0	0	0	0	0	0.7311	∼1	361.1	3.8	0.310	0.716	-0.274
GF1079	RM	0.341	0.573	0.156	-2.434	2.829	-0.021	-0.071	-0.151	∼0	0.5000	0.8	3.0	0.096	0.740	-6.436
GF1089	M	0.267	0.033	0	0	0	0	0	0	0.7311	0.9306	0.8	3.0	0.292	0.688	-0.064
GF1141	M	0.363	0.060	0	0	0	0	0	0	0.7311	0.9945	354.8	3.0	0.509	0.685	-0.344
GM1133	M	0.324	0.000	0	0	0	0	0	0	0.7311	∼1	47.4	22.3	0.495	0.698	-0.306
GF1087	RM	0.344	0.078	0.677	-1.405	-13.080	-36.366	-0.755	0.359	0.5200	0.8604	351.9	5.0	0.280	0.668	-1.030
GF1108	N	0.326	0.003	0	0	0	0	0	0	N/A	0	N/A	N/A	0.115	0.822	N/A
GF1143	RM	0.331	0.477	-0.020	-1.243	3.271	-0.065	-0.126	-0.196	0.0007	0.5000	299.1	6.5	0.060	0.781	-1.076
GM1147	M	0.464	0.000	0	0	0	0	0	0	0.7311	∼1	259.8	3.0	0.088	0.652	-0.512
GF1092	RM	0.241	0.000	0.189	-1.115	1.884	-0.096	-0.300	-0.250	0.0006	0.5005	334.6	30.8	0.314	0.602	-0.512
GF1146	M	0.319	0.159	0	0	0	0	0	0	0.7311	0.9885	346.8	3.0	0.097	0.701	-0.653

Table S2a: 95% lower confidence bounds for the "best model" (as identified by BIC) for each bear, calculated using likelihood profiling. Bears are listed in ascending order by number of GPS fixes. Gray text in the table indicates a parameter value that was fixed and not estimated for that model, and gray "N/A" values indicate parameters that are not influential in the "best model" for that bear. Parameter estimates for β_0 and β_d that are very close to but not exactly 0 or 1 are indicated as such with a "∼". See Table 1 for descriptions of each parameter.

ID	Model	ρ_{ns}	κ	β_1	β_2	β_3	β_4	β_5	β_6	β_0	β_d	μ	σ	λ	γ	α
GF1004	RM	0.409	0.603	0.252	0.510	1.809	0.003	0.090	0.044	0.2813	~1	356.4	12.8	0.558	0.788	-0.015
GM1046	RM	0.458	0.619	0.574	-0.267	1.861	0.000	0.112	0.053	0.9122	~1	363.0	17.9	0.451	0.801	-0.100
GF1008	R	0.474	0.628	0.248	-0.005	2.536	-0.013	0.015	-0.008	N/A	0	N/A	N/A	0.590	0.877	N/A
GF1086	RM	0.235	0.328	0.527	-0.498	5.036	-0.030	0.196	-0.097	0.0111	~1	1.2	3.3	0.462	0.800	-1.496
GF1016	M	0.326	0.332	0	0	0	0	0	0	0.7311	0.9933	4.6	4.0	0.652	0.792	0.112
GF1041	R	0.436	0.687	0.307	-0.336	3.840	-0.005	0.058	0.067	N/A	0	N/A	N/A	0.462	0.815	N/A
GF1107	R	0.279	0.377	0.452	0.798	3.263	0.002	0.126	0.201	N/A	0	N/A	N/A	0.517	0.787	N/A
GF1130	N	0.468	0.658	0	0	0	0	0	0	N/A	0	N/A	N/A	0.552	0.803	N/A
GF1005	R	0.716	0.464	0.332	0.428	5.696	-0.008	0.002	0.021	N/A	0	N/A	N/A	0.541	0.796	N/A
GF1096	RM	0.436	0.531	0.404	-0.806	3.578	-0.001	0.052	0.005	~1	0.6815	363.2	40.4	0.485	0.830	-0.079
GF1167	M	0.512	0.725	0	0	0	0	0	0	0.7311	~1	368.6	12.5	0.467	0.802	-0.126
GF1079	RM	0.419	0.894	0.358	-1.088	4.354	0.014	0.135	0.051	~1	~1	1.3	3.4	0.264	0.827	-0.946
GF1089	M	0.331	0.340	0	0	0	0	0	0	0.7311	0.9990	5.4	13.1	0.466	0.786	0.068
GF1141	M	0.487	0.484	0	0	0	0	0	0	0.7311	~1	365.0	31.7	0.697	0.817	-0.023
GM1133	M	0.435	0.288	0	0	0	0	0	0	0.7311	~1	61.0	33.9	0.690	0.829	-0.111
GF1087	RM	0.466	0.518	1.769	0.273	6.502	-0.053	-0.182	1.132	~1	~1	359.5	12.0	0.513	0.808	-0.478
GF1108	N	0.437	0.421	0	0	0	0	0	0	N/A	0	N/A	N/A	0.463	0.927	N/A
GF1143	RM	0.457	0.977	0.312	-0.256	6.609	-0.006	0.508	0.802	~1	~1	366.0	182.5	0.353	0.903	-0.119
GM1147	M	0.674	0.381	0	0	0	0	0	0	0.7311	~1	261.8	3.2	0.359	0.820	-0.440
GF1092	RM	0.370	0.108	0.830	3.508	4.001	-0.020	0.544	0.564	~1	~1	368.0	55.3	0.614	0.801	0.106
GF1146	M	0.543	0.960	0	0	0	0	0	0	0.7311	~1	353.2	7.0	0.543	0.908	-0.114

Table S2b: 95% upper confidence bounds for the "best model" (as identified by BIC) for each bear, calculated using likelihood profiling. Bears are listed in ascending order by number of GPS fixes. Gray text in the table indicates a parameter value that was fixed and not estimated for that model, and gray "N/A" values indicate parameters that are not influential in the "best model" for that bear. Parameter estimates for β_0 and β_d that are very close to but not exactly 0 or 1 are indicated as such with a "~". See Table 1 for descriptions of each parameter.

ID	Model	ρ_{ns}	κ	β_1	β_2	β_3	β_4	β_5	β_6	β_0	β_d	μ	σ	λ	γ	α
GF1004	RM	0.383	0.502	0.265	0.300	-0.265	-0.015	0.068	0.007	0.4360	~1	0.8	3.0	0.508	0.759	-1.611
GM1046	M	0.428	0.517	0	0	0	0	0	0	0.7311	~1	359.1	10.2	0.390	0.772	-0.126
GF1008	M	0.443	0.526	0	0	0	0	0	0	0.7311	~1	351.6	4.4	0.528	0.852	-0.133
GF1086	R	0.219	0.229	0.945	-0.792	3.529	-0.130	0.079	-0.120	N/A	0	N/A	N/A	0.403	0.770	N/A
GF1016	M	0.301	0.220	0	0	0	0	0	0	0.7311	0.9782	3.0	3.0	0.601	0.758	0.112
GF1041	N	0.405	0.578	0	0	0	0	0	0	N/A	0	N/A	N/A	0.399	0.785	N/A
GF1107	M	0.258	0.253	0	0	0	0	0	0	0.7311	~1	365.0	65.0	0.448	0.750	0.140
GF1130	RM	0.430	0.529	0.178	-0.110	-0.325	-0.051	0.002	-0.031	0.4415	~1	0.8	3.0	0.488	0.768	-1.762
GF1005	M	0.651	0.331	0	0	0	0	0	0	0.7311	0.9953	5.6	3.0	0.473	0.758	-0.256
GF1096	M	0.400	0.402	0	0	0	0	0	0	0.7311	~1	348.1	26.9	0.405	0.794	-0.079
GF1167	M	0.463	0.574	0	0	0	0	0	0	0.7311	~1	364.8	6.3	0.387	0.761	-0.126
GF1079	R	0.378	0.728	0.376	-2.161	2.508	0.012	-0.059	-0.141	N/A	0	N/A	N/A	0.171	0.786	N/A
GF1089	R	0.298	0.192	1.506	-0.783	1.243	-0.005	0.056	0.196	N/A	0	N/A	N/A	0.379	0.740	N/A
GF1141	M	0.419	0.529	0	0	0	0	0	0	0.7311	~1	365.0	11.9	0.606	0.754	-0.023
GM1133	M	0.374	0.080	0	0	0	0	0	0	0.7311	~1	54.5	26.7	0.595	0.768	-0.212
GF1087	M	0.400	0.295	0	0	0	0	0	0	0.7311	~1	357.9	9.2	0.396	0.744	-0.338
GF1108	N	0.376	0.211	0	0	0	0	0	0	N/A	0	N/A	N/A	0.273	0.880	N/A
GF1143	M	0.388	0.722	0	0	0	0	0	0	0.7311	~1	352.8	15.1	0.179	0.848	-0.119
GM1147	M	0.556	0.115	0	0	0	0	0	0	0.7311	~1	260.9	3.0	0.204	0.742	-0.477
GF1092	M	0.301	0.000	0	0	0	0	0	0	0.7311	~1	363.1	42.0	0.469	0.714	0.106
GF1146	M	0.412	0.549	0	0	0	0	0	0	0.7311	0.9998	350.0	3.0	0.286	0.819	-0.162

Table S3: Parameter estimates for the “best model” (as identified by BIC) for each bear with explicit expression of resource seasonality. Bears are listed in ascending order by number of GPS fixes. Note that the second letter of the bear ID indicates the sex of the individual. Gray text in the table indicates a parameter value that was fixed and not estimated for that model, and gray ”N/A” values indicate parameters that are not influential in the “best model” for that bear. Parameter estimates for β_0 and β_d that are very close to but not exactly 0 or 1 are indicated as such with a “$\sim$”.

References

Ducks Unlimited (2002). Lower Mackenzie River Delta, Northwest Territories Earth Cover Classification User's Guide. Technical report, Ducks Unlimited Inc., Rancho Cordova, USA.

Fischer, S. M. and Lewis, M. A. (2020). A robust and efficient algorithm to find profile likelihood confidence intervals. *ArXiv*, pages 1–18.

Kristensen, K., Nielsen, A., Berg, C. W., Skaug, H., and Bell, B. M. (2016). Tmb: Automatic differentiation and Laplace approximation. *Journal of Statistical Software*, 70(5).

Thompson, P. R., Derocher, A. E., Edwards, M. A., and Lewis, M. A. (2021). Detecting seasonal episodic-like spatio-temporal memory patterns using animal movement modelling. *Methods in Ecology and Evolution*, n/a(n/a).

Whoriskey, K., Auger-Methe, M., Albertsen, C. M., Whoriskey, F. G., Binder, T. R., Krueger, C. C., and Mills Flemming, J. (2017). A hidden Markov movement model for rapidly identifying behavioral states from animal tracks. *Ecology and Evolution*, 7(7):2112–2121.
